# Disruptions to safety and adaptations experienced by parents and caregivers who administered prescribed medicines to children at home: a systematic review using a framework synthesis

**DOI:** 10.3389/frhs.2026.1748195

**Published:** 2026-07-06

**Authors:** S. Morris, S. Pini, B. Fylan, F. Wilson, H. Faulkner, A. Vanzela, D. P. Alldred

**Affiliations:** 1 School of Healthcare, Worsley Building, University of Leeds, Leeds, United Kingdom; 2 Faculty of Medicine and Health, Worsley Building, University of Leeds, Leeds, United Kingdom; 3 School of Pharmacy and Medical Sciences, Faculty of Life Sciences, University of Bradford, Bradford, United Kingdom

**Keywords:** caregivers, domiciliary, framework synthesis, medication administration, parents, pediatrics, resilient healthcare, safety

## Abstract

**Background:**

Parents and caregivers are responsible for safely administering prescribed medicines to their children at home. This complex process relies on caregivers to be adaptive to any disruptions to this process that may diminish their ability to provide safe care to their children. Learning about the disruptions caregivers experience, alongside any adaptations to these disruptions, will inform improvements to healthcare systems. The aim of this review is to explore the experiences of disruptions to safety and any subsequent adaptations reported in research by parents and caregivers who administer prescribed medicines to children at home.

**Methods:**

This review followed the Cochrane-Campbell methodology for framework synthesis. Major healthcare databases (Embase, PyscINFO, CINAHL, Cochrane, PubMed) were searched to identify qualitative studies which reported the experiences of parents and caregivers who administered medicines to children at home. Quantitative studies were not included. The Joanna Briggs Institute Critical Appraisal Checklist for Qualitative Research was used to assess the quality of included studies. Extracted data from studies was organised and interpreted using the Moments of Resilience framework.

**Results:**

27,940 studies were identified and screened. Fifty-one studies were included in the review. The synthesis identified that caregivers experienced frequent disruptions from medicines, healthcare systems and family life that required adaptations to avoid causing harm to their children. Caregivers used a variety of short-term situated adaptations, that were supported by medium-term structural adaptations. However, these were not equally distributed across individuals and resources involved. There was a limited description of long-term systemic adaptations experienced by caregivers.

**Discussion:**

The use of adaptations by caregivers was well described and often involved significant effort from caregivers. The adaptive capability and capacity of caregivers was influenced by several factors such as family characteristics, access to resources and healthcare systems design. Future improvements to healthcare systems should reduce disruptions to avoid the need for adaptations entirely, alongside strengthening the ability of caregivers, professionals and healthcare systems to be adaptive to novel and unpredictable disruptions. Limitations include the variable quality of included studies, the lack of reporting of theoretical or cultural perspectives, and the influence of researchers on data and findings.

**Systematic Review Registration:**

https://www.crd.york.ac.uk/PROSPERO/view/CRD42024487154, PROSPERO #487154.

## 1 Background

The safe administration of prescribed medicines to children at home is a complex process. Administering a single dose safely requires caregivers to complete as many as twenty-three steps successfully each time a medicine is given (1), and failure at any point may result in harm. For example, overdosing causing toxicity (e.g., opioids and breathing difficulties) (2), or underdosing causing therapeutic failure (e.g., antibiotics and recurrent infections) (3). The nature of caring for a child is inherently variable and caregivers must navigate a wide range of disruptions to administer medicines safely.

Most research investigating the safety of medicines administered by parents and caregivers at home uses quantitative methods to audit deviations from procedures (4). This approach is often described as ‘*measure and manage’* in safety sciences (5). Deviations are referred to as administration errors and act as a surrogate for harm (6). A review has estimated that administration error rates range from 1.9 to 33% of all medicines given by caregivers at home (7).

The use of ‘*measure and manage’* has resulted in some improvements to safety at home. However, studies consistently report that approximately 5% of administration errors at home cause harm to children (8–10). The persistence of this harm may be related to limitations from the use of retrospective analysis of errors, which has been criticised for being reactive, time consuming, and restricting innovation (4, 11–13). This may explain the lack of interventions to improve safety in this area. Pictograms are currently the only proven intervention to support caregivers by reducing the risk of measurement errors during medication administration (14). The effectiveness of other interventions on safety, such as counselling from healthcare professionals remains unclear (15–17).

Public involvement work with caregivers of children has highlighted that many are able to use medicines safely despite the disruptions they experience (18). This corroborates other studies that have explored adult self-management of prescribed medicines at home (19–21). However, little work has been done to explore the experiences of caregivers of children to learn how they proactively overcome disruptions whilst maintaining safety. Recent developments in safety theory, such as the concept of resilient healthcare, can guide this exploration (22). ‘Resilient Healthcare’ is defined as the “*capacity to adapt to challenges and changes at different system levels in order to maintain high quality care*” (23).

Studying the solutions to disruptions, such as adaptations, can provide a more effective enquiry about those disruptions as well as identifying potential solutions, than merely studying disruptions by themselves (11). Further guidance on complex intervention development also highlights the importance of exploring context and refining theory when developing interventions to improve healthcare (24).

Therefore, the aim of this review is to explore the experiences of disruptions to safety and adaptations reported in research by parents and caregivers who administer medicines safely to children at home.

## 2 Methods

A framework synthesis was conducted following the Cochrane-Campbell Handbook for Qualitative Framework Synthesis (25), which involved five stages:

- familiarisation (from developing the review protocol and searches),
- thematic framework identification (from reading the current safety literature),
- indexing (from identifying relevant text within studies and assigning an appropriate category within the framework),
- charting (from organising extracted data according to category),
- mapping and interpretation (from reviewing dataset and write-up).

Moments of Resilience (26) was identified as a suitable safety framework for this review within resilient healthcare that describes “*the scale and nature of organisational activity that unfolds around a disruption*” (26). Within the framework, disruptions are defined as a type of variation, of which there are two sub-types:

- *Fluctuations* – variations in everyday life that do not pose an immediate threat to situated control.
- *Disruptions* – variations that require action to avoid the loss of situated control.
- Variations that escalate to be perceived as disruptive events then provoke adaptive responses across three broad categories of time and scale:
- *Situated* – the immediate application of existing resources at or close to the frontline in response to disruptions.
- *Structural* – the restructure of resources and situated practices to handle larger disruptions, or where situated adaptations are insufficient.
- *Systemic* – the wider reform of resources that support situated practices where situated or structural adaptations are insufficient.

These definitions were used to guide the indexing, charting, mapping and interpretation stages. The full review protocol is published in *Systematic Reviews* (27), and registered with International Prospective Register of Systematic Reviews database #487154. The manuscript was written according to Enhancing transparency in reporting the synthesis of qualitative research: ENTREQ (refer to Supplementary Materials for checklist) (28).

### 2.1 Search strategy

An iterative search strategy was conducted across five major healthcare databases (Embase, PyscINFO, CINAHL, Cochrane and PubMed) (29). The strategy was structured using Population, Exposure, and Outcome concepts (30). These were: parents and caregivers (population), administration of medicines (exposure), and the safe administration of medicines in the home (outcome). Search terms were developed using a set of seed references (8, 31–33) to ensure sensitivity of searches (34). No filters or criteria were applied to search results. Searches were supplemented by manual footnote chasing by a reviewer (SM) (35). A full search strategy is provided in the Supplementary Materials.

### 2.2 Inclusion criteria

Studies were included if they:

- Reported empirical research published in peer reviewed journals.
- Used qualitative methods, or mixed methods where the qualitative data component could be clearly extracted.
- Included data related to the administration of prescribed medicines to children (defined as <18 years of age) by parents and caregivers at home.

### 2.3 Study screening methods

Database searches were transferred to Rayyan® and duplicates removed. Studies were screened for relevance by title and abstract with the first 10% screened independently by two reviewers to check performance of searches and screening steps (SM and FW). The remaining studies were screened by one reviewer (SM). Studies that passed the first screen had their full text assessed for inclusion by two independent reviewers (SM and FW) who discussed any disagreements until agreement was reached.

Methodological quality of included studies was assessed using the Joanna Briggs Institute (JBI) Critical Appraisal Checklist for Qualitative Research by two independent reviewers (SM and FW) (34). No cut-off was used for inclusion from the quality assessment.

### 2.4 Data extraction and analysis

Studies published in languages other than English were translated using ChatGPT (OpenAI Inc., US) with the translations refined by reviewers fluent in those languages (HF and AV).

Studies were read line by line and data were coded from the results and discussion sections using Microsoft Edge PDF editor (Microsoft, US). Reviewers sought to identify experiences as described by quotations, or statements by researchers that were a summation of a collection of experiences. All studies had coded data extracted by at least one reviewer (SM), and a subset were also co-extracted by multiple reviewers (SM with HF/FW/HN/RA/AH/EM/NM).

Microsoft Excel (Microsoft, US) was used to organise extracted data according to the elements within the Moments of Resilience theory. These were: fluctuations, disruptions, situated, structural and systemic. Additional data were extracted regarding study characteristics.

### 2.5 Patient involvement

Public involvement was conducted throughout this research. The research question was identified during a focus group with parents who gave medicines at home. Further input has been sought with the selection of theoretical perspective, data coding and extraction, and interpretation. This was undertaken with a parent advisory group, individual public advisors, and engagement with members of the public at community events. Refer to Supplementary materials for more descriptions of public involvement activities.

## 3 Results

### 3.1 Study selection results

The database searches (March 2024) retrieved 28,309 results, 5,820 were duplicates and removed with 22,489 studies screened for eligibility. The first 2,251 (10%) studies were screen independently by two reviewers (SM and FW), the remaining were screened by one reviewer (SM).

**Figure 1:**
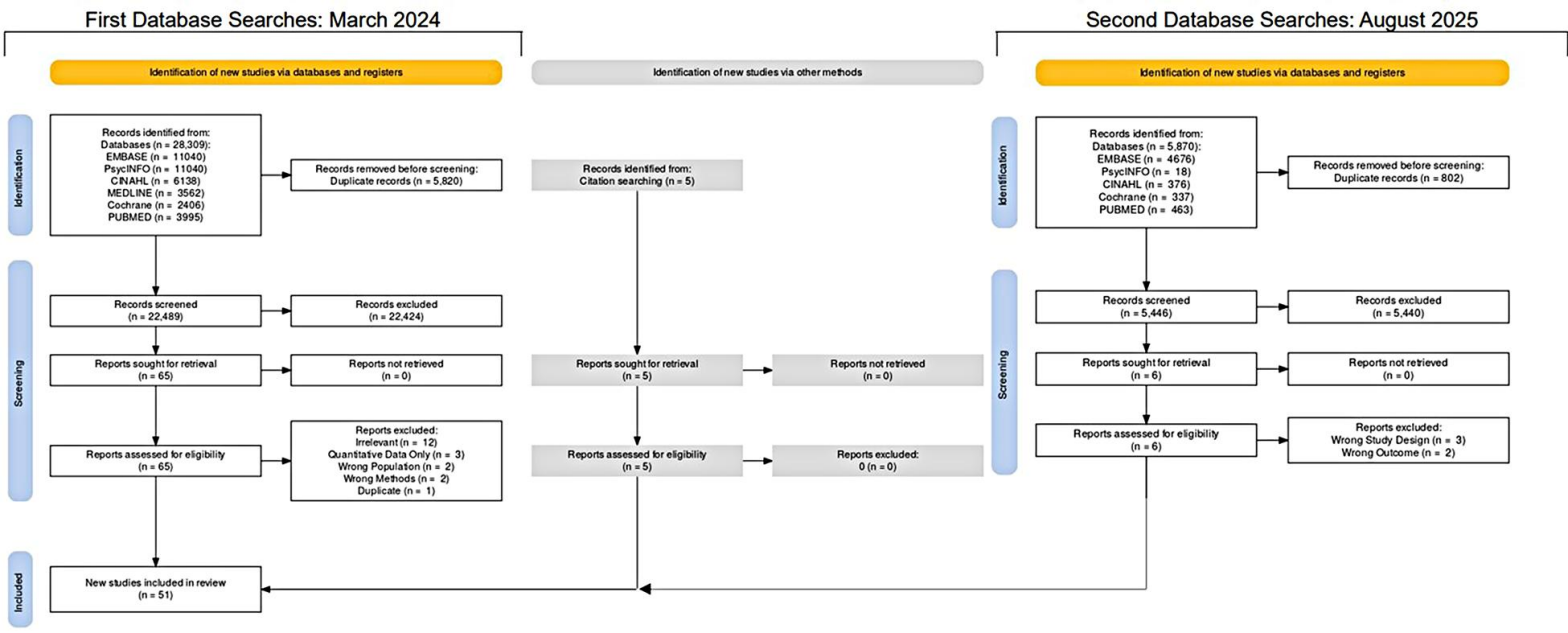
PRISMA diagram.

Sixty-five studies were eligible for full text assessment. Twenty studies were excluded (refer to PRIMSA diagram - Figure 1 for reasons for exclusion). During the process of this review, an additional five studies were identified from footnote chasing. The search was re-run in August 2025 following the steps outlined above. This resulted in an additional 5,446 studies, of which one study was included (refer to PRIMSA diagram - Figure 1).

Therefore, this review included fifty-one studies. Nine of which were co-extracted by two reviewers (SM with HF-public advisor/FW/HN/RA-public advisor/AH/EM/NM), the remaining studies were extracted by one reviewer (SM).

### 3.2 Study characteristics

Included studies represented a wide and diverse range of research. All studies except 3 were published in English: 1 in French (36) and 2 in Portuguese (37, 38). A full list of the included study characteristics is provided in Table 1.

**Table 1:** Characteristics of included studies.

Lead Author and Date	Country and Language(s) spoken by participants	Clinical setting and research aim	Study Design, Qualitative Component(s) and method of analysis	Sample Size, Characteristics and Profession of the Primary Author	Method of Qualitative Data Collection	Main finding(s) and any further work to develop or test complex interventions or treatments
Akkawi, 2023 (39)	Sweden/Swedish	Haematology and Oncology To describe the experiences of parents handling oral anticancer drugs in the home setting.	Qualitative Semi-structured interviews Content Analysis	18 parents Pharmacy.	Interview in-person using hospital clinic room.	Parents need to be provided with accurate, timely, nonconflicting and repeated information—in different forms and in their mother tongue—on how to handle oral anticancer drugs at home. Research used to inform development of education materials for parents. (40)
Arnold, 2018 (40)	USA/Spanish & English	Respiratory To assess differences among child, caregiver and clinician views toward asthma controller medication barriers.	Mixed methods Survey & Questionnaire Semi-structured Interviews Thematic Analysis	26 triads (parent, patient and clinician) Primary care physician	Interview via in-person clinic visit or via telephone at home.	There was significant disagreement among children, caregivers, and clinicians regarding barriers to daily use of asthma medications. To tailor asthma management conversations, clinicians should understand family-specific barriers and child–caregiver disagreements. No subsequent research towards complex intervention development or testing published to date.
Aston, 2018 (34)	UK/English	Cross-specialty To explore the treatment related experiences when children and young people take regular prescribed medication.	Qualitative Semi-structured interviews Thematic Analysis	24 families. Pharmacy.	Interview in-person during inpatient hospital stay.	Patients and parents experience many challenges with children's medication. Individualised treatment options should be considered. No subsequent research towards complex intervention development or testing published to date.
Benleulmi, 2019 (36)	France/French	Rheumatology To access the lived experiences of parents to understand how they organise, proceed and mentally cope with injecting their children.	Qualitative Explicative Interviews Thematic Analysis	10 parents (8 mothers and 2 fathers). Not specified.	Interview	Injecting medication into a child is not easy for a parent, even when the technique is mastered. No subsequent research towards complex intervention development or testing published to date.
Bracken, 2023 (32)	UK & Ireland/English	Neonatology To explore parents’ experiences of medicine management of their baby post-discharge from neonatal units.	Qualitative Focus groups and semi-structured interviews Thematic Analysis	14 mothers and 3 fathers Pharmacy	Focus groups and interviews	Despite the challenges they faced, parents developed strategies for safely and reliably managing medicines administration and they assimilated knowledge, built their confidence and achieved a capability in medicines administration. This research has been used to develop medicines administration resources to support future parents. (42)
Camiré-Bernier, 2021 (41)	Canada/French	Haematology and Oncology To describe the parents’ experiences of home based mercaptopurine and dexamethasone prescribed to a child diagnosed with leukaemia	Qualitative Semi-structured interviews Thematic Analysis	13 parents Pharmacy	Interview via in-person clinic visit or via telephone at home.	Supportive interventions should consider the family as a whole and their needs should be regularly monitored. Specific attention should be paid to the development and maintenance of a routine, to the parental burden, and to the emotional impact. No subsequent research towards complex intervention development or testing published to date.
Carroll, 2023 (42)	USA/Spanish & English	Cross-specialty To explore the perspectives of clinicians and caregivers regarding discharge medication counselling.	Qualitative Focus groups and interviews Thematic Analysis	17 caregivers Physician.	Virtual (via video calls) at home	Interventions should use a health literacy universal precautions approach-written materials with plain language and pictures and verbal counselling with teach-back and show-back. This research informed a randomised control trial of a complex intervention to reduce measurement errors by caregivers. (45)
Chew, 2020 (43)	Malaysia/Malay & English	Cross-specialty To explore issues related to out of hospital mediation safety among the paediatric outpatients in Malaysia from a caregivers’ perspective.	Qualitative Semi-structured interviews Thematic Analysis	15 mothers. Pharmacy.	Interview in-person using hospital clinic room.	Caregivers recommended the use of written instructions and a diary as potential effective strategies to improve the out-of-hospital medication safety in children. No subsequent research towards complex intervention development or testing published to date.
Coelho, 2015 (44)	Brazil/Portuguese	Medical complexity To identify the feasibility of home care and the difficulties of mothers who deliver this care to children with special health needs.	Qualitative Semi-structured interviews Thematic Analysis	10 mothers Not specified.	Interview in-person using hospital ward.	Mothers should be better prepared for the transition from hospital to home. Healthcare systems should reorganise to provide more care in community. This could reduce families dependence on hospitals. The expansion of social networks between families should be further explored. No subsequent research towards complex intervention development or testing published to date.
Doyle, 2022 (33)	Ireland/English	Medical complexity. To explore mothers’ lived experience of giving medicines to children with severe and profound intellectual disabilities.	Qualitative Semi-structured interviews Thematic Analysis	15 mothers Nursing.	Interview in person, location not specified	The experiences of mothers who give medicines to children with severe intellectual disabilities is a relentless and challenging one. It is invisible as an element of care in professional discourse. Through addressing the gap in understanding, it may be useful in developing care for mothers and children with severe and profound intellectual disabilities. No subsequent research towards complex intervention development or testing published to date.
Dy, 2018 (45)	USA/English	Respiratory To better understand the perceptions of African American caregivers for short acting beta-agonist inhalers.	Qualitative Focus groups Inductive Content Analysis	26 parents Physician.	Focus group in person using community venues.	Caregivers suggested that education on symptom recognition and close communication between physician and patient would facilitate the implementation of an intervention. No subsequent research towards complex intervention development or testing published to date.
Emgård, 2022 (46)	Tanzania/Kiswahili	Infectious diseases To describe mothers’ experiences of antibiotic use in under-five children.	Qualitative Focus groups Phenomenological approach (nota bene [n.b.] results presented as themes)	54 mothers Physician.	Focus group in person using public health centre.	Antibiotic misuse could be reduced by improving structures such as the healthcare system, socioeconomic inequalities and promoting gender equality both in the household and in the public arena. Equipping community health workers to support Tanzanian women in appropriate healthcare seeking for their children, may be a feasible target for intervention. No subsequent research towards complex intervention development or testing published to date.
Fang, 2024 (47)	China/Not specified	Gastroenterology To investigate the experiences of caregivers of infants undergoing home reflux enemas.	Qualitative Semi-structured interviews Phenomenological approach (Colaizzi's 7 step analysis method)	7 mothers, 2 fathers, 1 grandmother and 2 grandfathers. Nursing.	Interview via telephone call at home.	Recommendations include positive psychological interventions using the PERMA model (Positive Emotion, Engagement, Relationships, Meaning, and Accomplishment.) alongside “collaborative nursing”. This will provide caregivers with professional knowledge, address their pressures and needs, and promote their well-being while enhancing nursing abilities. No subsequent research towards complex intervention development or testing published to date.
Findlater, 2022 (48)	USA/English	Neonatology To explore the parent experiences with the Meds to Beds programme.	Qualitative Semi-structured interviews Inductive Content Analysis	11 parents. Pharmacy.	Interview via telephone call at home.	The intervention was helpful to increasing parental confidence and knowledge around medications and reducing stress around the acquisition of medications for home. It also improved relationships between families and professionals that allowed a tailored approach to coordinating the needs of families. No subsequent research towards complex intervention development or testing published to date.
Flankegård, 2020 (49)	Sweden/Not specified	Gastroenterology To explore the lived experiences of giving pharmacological childhood constipation treatment at home.	Qualitative Semi-structured interviews Phenomenological approach (reflective lifeworld research approach)	10 mothers and 5 fathers. Nursing.	Interview via home, hospital or private location as chosen by participants	The findings point to the importance of supporting parents in treatment situations. Healthcare providers need to treat children with constipation with greater focus and more prompt management to prevent these families from lingering longer than necessary in the healthcare system. No subsequent research towards complex intervention development or testing published to date.
Flynn, 2019 (50)	South Africa/Tshivenda or English	Infectious diseases To understand the experiences of administering medication to infants from the perspective of infant caretakers and rural health workers.	Qualitative Semi-structured interviews Phenomenological approach (Tesch's method)	30 mothers, 5 grandmothers and 9 health workers No specified.	Interview via in-person home visit or using hospital clinic room.	Reuse of medication in the home and home hygiene practices surrounding infant medication administration are areas of potential future research. No subsequent research towards complex intervention development or testing published to date.
Forsner, 2014 (51)	Sweden/Not specified	Endocrinology To determine parents’ experiences of caring for a child younger than two years old with diabetes mellitus being treated with continuous subcutaneous insulin infusion.	Qualitative Semi-structured interviews (longitudinal) Qualitative content analysis	3 dyads (mother and father) Nursing.	Interview via in-person home visit or using hospital clinic room.	Parents of infants with diabetes are in great need of support in order to manage the disease and infusion technology. Educating someone close to the family could be a valuable intervention. No subsequent research towards complex intervention development or testing published to date.
Gedaly-Duff, 1994 (52)	USA/English	Surgery To describe mothers’ experiences in identifying and managing their children's acute pain associated with surgery.	Qualitative Semi-structured interviews Grounded theory	7 mothers Nursing.	Serial interviews in person: 1st interview in hospital, 2nd & 3rd at home (2 mothers opted for telephone interview at home)	Future research needs to describe how families build on past experiences and the impact of children's developmental factors on family pain management activities. In addition, designing studies that examine the family as a unit (total family) will increase our understanding of the interactive effects of family and pain expression in Children. No subsequent research towards complex intervention development or testing published to date.
Gilmore, 2022 (53)	Australia/English	Haematology To explore the educational needs of parents who infants require enoxaparin anticoagulation after discharge	Qualitative Focus group Thematic Analysis	5 dyads (mother and farther), 1 single mother. Not specified.	Not specified	The availability of an educational resource for families to refer to once discharged, as well as ongoing communication with the treating medical team is vital. No subsequent research towards complex intervention development or testing published to date.
Gomes, 2019 (54)	Brazil/Portuguese	Haematology and oncology To know the care taken by the family caregiver of the child submitted to hematopoietic stem cell transplantation.	Qualitative Semi-structured interviews Grounded Theory (Strauss and Corbin method)	36 caregivers. Not specified.	Interviews in person via hospital and transition homes.	Healthcare teams should understand the different aspects in which the caregiver acts in the care of the child. This understanding will allow for guidance and preparation of home care that are effective and directed to the needs of the patient and their family No subsequent research towards complex intervention development or testing published to date.
Hoegy, 2019 (55)	France/French	Haematology and oncology To provide an understanding of medication adherence after paediatric allogeneic stem cell transplantation.	Qualitative Semi-structured interviews General Inductive Approach	12 mothers and 3 fathers. Physician.	Interviews in person using hospital clinic room.	Caregivers expressed the need to take into consideration the family entity. They would like also to receive earlier information from healthcare providers before hospital discharge. Those needs were not always identified by healthcare providers. Authors mention developing support at their institution but not published.
King, 2018 (56)	Malawi/Chichewa	Infectious diseases To investigate reasons for adherence and non-adherence in children diagnosed and treated in the community with fast breathing pneumonia and rural Malawi.	Qualitative Focus groups Framework Analysis	24 caregivers. Academic.	Interviews in a community setting (not specified)	Considerable barriers were described within this rural low-resource setting, such as the effort preparing and administering medication, community pressures to share drugs and potential complexity of regimens. No subsequent research towards complex intervention development or testing published to date.
Klok, 2014 (57)	Netherlands/Not specified	Respiratory To explore factors that contribute to persistent non-adherence to inhaled corticosteroids.	Qualitative Semi-structured interviews Standard methodology for qualitative studies	17 fathers and 25 mothers Physician.	Interview in-person using home visit	This study has identified modifiable barriers to adherence, future studies are needed to investigate whether interventions around these barriers are needed to increase the effectiveness of asthma care programs. No subsequent research towards complex intervention development or testing published to date.
Kremeike, 2015 (58)	German/German	Haematology and oncology To analyse factors influencing the adherence of patients with leukaemia undergoing maintenance therapy.	Mixed methods Semi-structured interviews Content Analysis	22 mothers, 4 fathers and 1 couple (not specified). Not specified.	Interview in-person using home visit or university clinic.	Parents' information on drug therapy should be more consistent and the pharmaceutical formulations have to be adapted to patients' needs to improve adherence. No subsequent research towards complex intervention development or testing published to date.
Lakhanpaul, 2017 (59)	UK/English and multiple South Asian languages (not specified)	Respiratory To explore perceptions and experiences of asthma and asthma management in British South Asian and White British families.	Qualitative Semi-structured interviews Thematic Analysis	42 mothers and 19 fathers and 5 caregivers. Physician.	Interview in-person using home visit	Culturally sensitive, holistic and collaboratively designed interventions are needed. Improved communication support for families with lower proficiency in English is required. Healthcare professionals need to make greater efforts to check families' understandings of symptoms, use of medications and accessing help. No subsequent research towards complex intervention development or testing published to date.
Law, 2020 (60)	Online/English	Ophthalmology To explore parents’ experiences of administering eye drops to their children.	Qualitative Online message boards and blog posts Thematic Analysis	464 unique posts. Psychologist.	Online	The advice given to parents needs to go beyond the instillation of the eye drops, and include advice on child restraint, distraction techniques and allaying distress. Forewarned of the potential difficulties and provided with coping strategies parents can employ when the child resists, could alleviate their own and their child's distress. No subsequent research towards complex intervention development or testing published to date.
Longard, 2016 (61)	Canada/English	Surgery To explore parents’ experience of managing their child's post operative pain at home.	Qualitative Semi-structured interviews Content Analysis	9 mothers and 1 father. Psychologist.	Interview via telephone call	The results of this study may aid in the design of interventions that will support parents when managing their child's postoperative pain at home and thus improve children's experiences. No subsequent research towards complex intervention development or testing published to date.
McCall, 2017 (62)	Ireland/English	Haematology and oncology To evaluate a nationwide initiative to educate and support parents to administer chemotherapy to their child in their home.	Mixed methods Questionnaire Content Analysis	108 parents. Nursing.	Questionnaire posted to participants and returned via post, or to hospital clinic	: An important feature of the program is the partnership approach, which ensures that parents’ decision to enter the program is informed, appropriate for their situation, and centred on the needs of the child. No subsequent research towards complex intervention development or testing published to date.
Min, 2023 (63)	USA/English	To describe strategies developed or adapted by family caregivers managing medications for their children and with special healthcare needs. Medical complexity	Qualitative Semi-structured interviews Thematic Analysis	20 mothers (14 biological, 6 adoptive). Not specified.	Interviews using virtual video call	These findings can inform current clinical practice through improved awareness of different strategies employed by caregivers and lay a foundation to develop interventions designed to support caregiver mediated medication management. No subsequent research towards complex intervention development or testing published to date.
Monnerat, 2016 (64)	Brazil/Not Specified	Medical complexity To identify the doubts of relatives of children with special health care needs related to the continued use of drugs.	Qualitative Semi-structured interviews Content Analysis	13 caregivers (8 mothers, 2 grandparents, 2 sisters and 1 aunt). Nursing.	Interview in-person using private room in hospital.	The conversation wheel is an important strategy for preparing the relatives of CRIANES with medical care demand for their hospital discharge. No subsequent research towards complex intervention development or testing published to date.
Ndou, 2022 (65)	South Africa/English or local languages (not specified)	Endocrinology To explore challenges experienced by caregivers during the provision of care to type 1 diabetic children.	Qualitative Semi-structured interviews Tesch's open coding method	15 caregivers. Nursing.	Interview in-person using home visit	It is imperative to educate the caregivers on the care of children with type 1 diabetes mellitus for them to be competent and knowledgeable in assisting their diabetic children at home. No subsequent research towards complex intervention development or testing published to date.
Okido, 2016 (66)	Brazil/Portuguese	Medical complexity To understand the experience of mothers of technology-dependent children as regards pharmaceutical care.	Qualitative Semi-structured interviews Thematic Analysis	12 mothers. Not specified.	Interview in-person using home visit	Pharmaceutical care is a daily challenge expressed in maternal overload and difficulty accessing the drugs, made worse by failures in the care network and coordinated care. No subsequent research towards complex intervention development or testing published to date.
Orford, 2023 (67)	Australia/English	Haematology and oncology To describe the implementation process and evaluation of a home intravenous hydration program for children with cancer.	Qualitative Semi-structured interviews Thematic Analysis	11 caregivers. Nursing.	Interview via telephone call at home	When adequately trained and well supported, parents highly value providing home-based care to their children. This offers opportunities to improve experiences and outcomes for children and families as well as reduce costs to health services, achieving clinical impact without reducing safety. No subsequent research towards complex intervention development or testing published to date.
Phillips, 2023 (68)	USA/English	Haematology To explore perspectives of decision making for opioid medications in sickle cell disease.	Qualitative Semi-structured interviews Content Analysis	10 caregivers. Nursing.	Interview in-person using private room in hospital.	opioid management for pain in SCD is important yet complex and requires collaboration among patients, families, and providers. Elements of patient and caregiver decision-making identified in this study may be applied to shared decision-making strategies in the clinical setting and future study. No subsequent research towards complex intervention development or testing published to date.
Powers, 2020 (69)	USA/English & Spanish	Haematology To characterise barriers to and facilitators of successful iron therapy in young children with iron deficiency anaemia from an in-depth parental perspective.	Mixed methods Semi-structured interviews Thematic Analysis	20 caregivers. Physician.	Interview in person using hospital outpatient clinic room.	Our findings support the need for interventions designed to promote oral iron adherence in children with IDA. Rather than focusing on knowledge content related to IDA, interventions should aim to increase parental motivation by emphasizing the health benefits of adhering to iron therapy and avoiding more invasive interventions. No subsequent research towards complex intervention development or testing published to date.
Rahimi, 2019 (70)	Uganda/English & Lunyole	Infectious diseases To discuss the challenges experience by heads of households’ and caregivers’ when seeking health care for their children five years or under with fever presumed to be malaria.	Qualitative Focus groups Thematic Analysis	60 participants. Physician.	In person focus groups using community centres	Future interventions may need to look beyond the public health system to improve case management of childhood malaria at the community level in rural districts such as Butaleja. No subsequent research towards complex intervention development or testing published to date.
Ramos, 2017 (71)	Brazil/Not specified	Medical complexity To analyse the maternal perceptions about paternal care and how this care is made effective in practical actions in the care of the child/adolescent with chronic disease in the family routine.	Qualitative Semi-structured interviews Thematic Analysis	10 caregivers. Not specified.	Interview in-person using hospital outpatient clinic room	Increasing the fathers’ participation and importance of sharing responsibilities was evidenced so that the mothers feel more relieved and less burdened as to the responsibilities assumed towards the illness of the child. Guidance should be given to additional family members to minimise the load on the main caregiver. No subsequent research towards complex intervention development or testing published to date.
Ribeiro, 2022 (72)	Brazil/Portuguese	Gastroenterology To describe home care practices performed by family members for maintaining the life of children with gastrostomy.	Mixed methods Workshops Lexical Analysis	10 caregivers. Nursing.	Workshops in person using hospital outpatient clinic room.	Family caregivers used strategies to maintain the gastrostomy and acquired new knowledge in this field such as stoma care, food administration, and medication administration. No subsequent research towards complex intervention development or testing published to date.
Santer, 2012 (84)	UK/English	Dermatology To explore carers of children with eczema views and experiences of the condition and its management.	Qualitative Semi-structured interviews Thematic Analysis	31 parents (28 mothers and 3 fathers). Primary care physician.	Interview in-person using home visit or GP surgery room	Poor concordance with treatments seems unsurprising in the presence of such dissonance between carers' and healthcare providers' agendas. Acknowledging the impact of the condition, greater attention to how key messages are delivered and addressing carers' treatment beliefs are likely to improve engagement with effective self-care. Several trials evaluating management strategies for eczema. (77, 78)
Schmitd, 2012 (37)	Brazil/Portuguese	Neonatology To understand the experience of mothers of premature infants in home care after hospital discharge.	Qualitative Semi-structured interviews Bardin's methodological framework	4 mothers. Nursing.	Interview in-person using home visit	To ensure greater safety for both parents and infants, outreach teams should be trained regarding premature neonates and be available after discharge to families. No subsequent research towards complex intervention development or testing published to date.
Silveira, 2022 (38)	Brazil/Portuguese	Medical complexity To describe the daily challenges encountered by family caregivers in providing home care to children with special health needs	Qualitative Semi-structured interviews Content Analysis	15 caregivers (13 mothers and 2 grandmothers). Nursing.	Interview in-person using home visit	The study suggests that these families should receive support and guidance from health services to help minimize the daily care challenges within the home. No subsequent research towards complex intervention development or testing published to date.
Silver-Rodrigues, 2018 (74)	Brazil/Portuguese	Haematology To describe the experiences of mothers of children with sickle cell anaemia with regards to home based medication therapy.	Qualitative Semi-structured interviews Content Analysis	10 mothers. Nursing.	Interview in-person using private room in hospital.	Nurses need to act in advising the mothers for safe and correct drug administration at home, with a view to reducing complications related to the absorption of the drug, therapeutic suppression and caregivers’ undue exposure to the drug. No subsequent research towards complex intervention development or testing published to date.
Slatter, 2004 (75)	UK/English	Respiratory. To investigate the perspectives of parents of children with cystic fibrosis regarding their roles in managing medication.	Qualitative Semi-structured interviews Thematic Analysis	15 mothers and 2 fathers. Pharmacy.	Interview in-person using home visit.	The involvement of healthcare professionals across primary, secondary and tertiary care sectors in supporting parents in all aspects of medication management, including the development of strategies for transferring the responsibility for medication to their children, must be improved. No subsequent research towards complex intervention development or testing published to date.
Souza, 2020 (76)	Brazil/Portuguese	Endocrinology To learn family caregivers’ perspectives and experiences regarding home care given to children and adolescents with type 1 diabetes mellitus.	Qualitative Semi-structured interviews Thematic Analysis	10 mothers and 1 father. Nursing.	Interview in-person using home visit.	These results reinforce the importance of intersectoral actions in the follow-up of families, recognising that, during childhood and adolescence, professionals working in the school environment can be very important in managing treatment. No subsequent research towards complex intervention development or testing published to date.
Sutton, 2022 (77)	UK/English	Dermatology To identify parents’ and children's experiences of emollient use.	Qualitative Semi-structured interviews Thematic Analysis	44 parents. Academic.	Interview at home using in-person visit or telephone call	Future research could evaluate decision aids and/or tester pots of different types of emollient, which could enable clinicians and parents/children to work collaboratively to identify the best emollient for them. Several trials evaluating management strategies for eczema. (93, 94)
Talegaonkar, 2023 (78)	India/Not specified	Infectious disease. To understand the use and challenges of traditional devices and assess the need of innovative administration devices for liquid orals.	Mixed methods Questionnaire and Workshops Interpretive Descriptive Approach	271 caregivers. Pharmacy.	In person workshops, venues not described.	This study highlights key issues with the use of appropriate administration devices for correct and accurate dosing in children that remain unresolved and prevalent in India. This study reflects on the needs of the target community which should facilitate the development of locally sustainable solutions to improve the administration of medicines in children in India. No subsequent research towards complex intervention development or testing published to date.
Tang, 2022 (79)	Canada/English	Haematology and oncology. To describe the experiences and perspectives of parents of paediatrics patients with leukaemia regarding oral chemotherapy administration during maintenance therapy.	Mixed methods Semi-structured interviews Descriptive Analysis	23 parents. Pharmacy.	Interview at home using in-person visit or telephone call	Oral chemotherapy administration during leukemia maintenance therapy was hard for some parents. Identification of these parents and discussion of strategies to facilitate adherence to oral chemotherapy regimens may optimize patient outcomes. No subsequent research towards complex intervention development or testing published to date.
Tran, 2017 (80)	Vietnam/Vietnamese	Infectious disease. To explore and measure caretakers’ barriers in order to improve paediatric antiretroviral adherence in the future.	Mixed methods Focus groups and semi-structured interviews Content Analysis	53 participants (8 men and 45 women), n.b. 31 were biological parents. Physician.	Interviews and workshops in person using hospital outpatient clinic room.	Family residence, caretaker's education level and job were considered as the key factors determining caretakers' barriers related to financial burden and stigma. These findings may be important for policy makers and researchers in order to develop effective interventions regarding to caretakers' burdens and associated factors. Authors mention a tool for nurses to identify barriers to adherence. No subsequent publications identified.
Tulczek, 2022 (81)	USA/English & Spanish	Respiratory To increase understanding of parents’ experiences managing the needs of their children with cystic fibrosis.	Qualitative Questionnaires Grounded Dimensional Analysis	80 participants. Nursing.	Questionnaire completed online or via telephone	Parental expertise represents an essential aspect of family adaptation and child health outcomes. Care teams might critically self-examine their expectations and collaborate with parents to develop more reasonable expectations. No subsequent research towards complex intervention development or testing published to date.
van Uum, 2019 (82)	Netherland/Not specified	Infectious diseases. To understand parents’ views and expectation of pain management in acute otitis media in children.	Qualitative Semi-structured interviews Thematic Analysis	14 parents (9 mothers and 5 fathers). Primary care physician.	Interview at home, method not specified	It is important that primary care physicians are aware of parents’ lack of understanding of the role of pain medication in managing acute otitis media, and that they address this during the consultation. No subsequent research towards complex intervention development or testing published to date.
Zupanec 2025 (97)	Canada and USA/English, Spanish & Ukrainian	Haematology and oncology To describe the family caregiver experience of caring for their child receiving blinatumomab at home.	Qualitative Semi-structured interviews Thematic Analysis	20 parents (15 mothers and 5 fathers)	Interview at home using in-person visit or telephone call	Caregivers can confidently manage home-based continuous infusions with anticipatory guidance and support, and with attention paid to context-related modifiers to care. Caregiver insights should be reflected in the principles that become the basis of future leukaemia clinical trials and care. This study is part of a wider evaluation of the implementation of home base chemotherapy treatment which is influencing guidelines and policy. (87)

The methods of data collection within studies included workshops, semi-structured interviews, online message boards and focus groups with the predominant approach to data analysis being thematic analysis.

### 3.3 Appraisal results

The quality of the research varied (see Supplementary Materials for quality assessment). No studies were excluded based on quality assessment. Studies were generally consistent with their application of methodologies, representations of participants’ data and had received ethical review.

### 3.4 Framework synthesis output

For the first part of this synthesis, data are organised under each of the domains of the framework: fluctuations, disruptions, situated adaptations, structural adaptations and systemic adaptations.

Further analysis was then conducted to understand the process that adaptations are formed and implemented, and how the design of research influenced the description of disruptions and adaptations by parents and caregivers.

**Figure 2:**
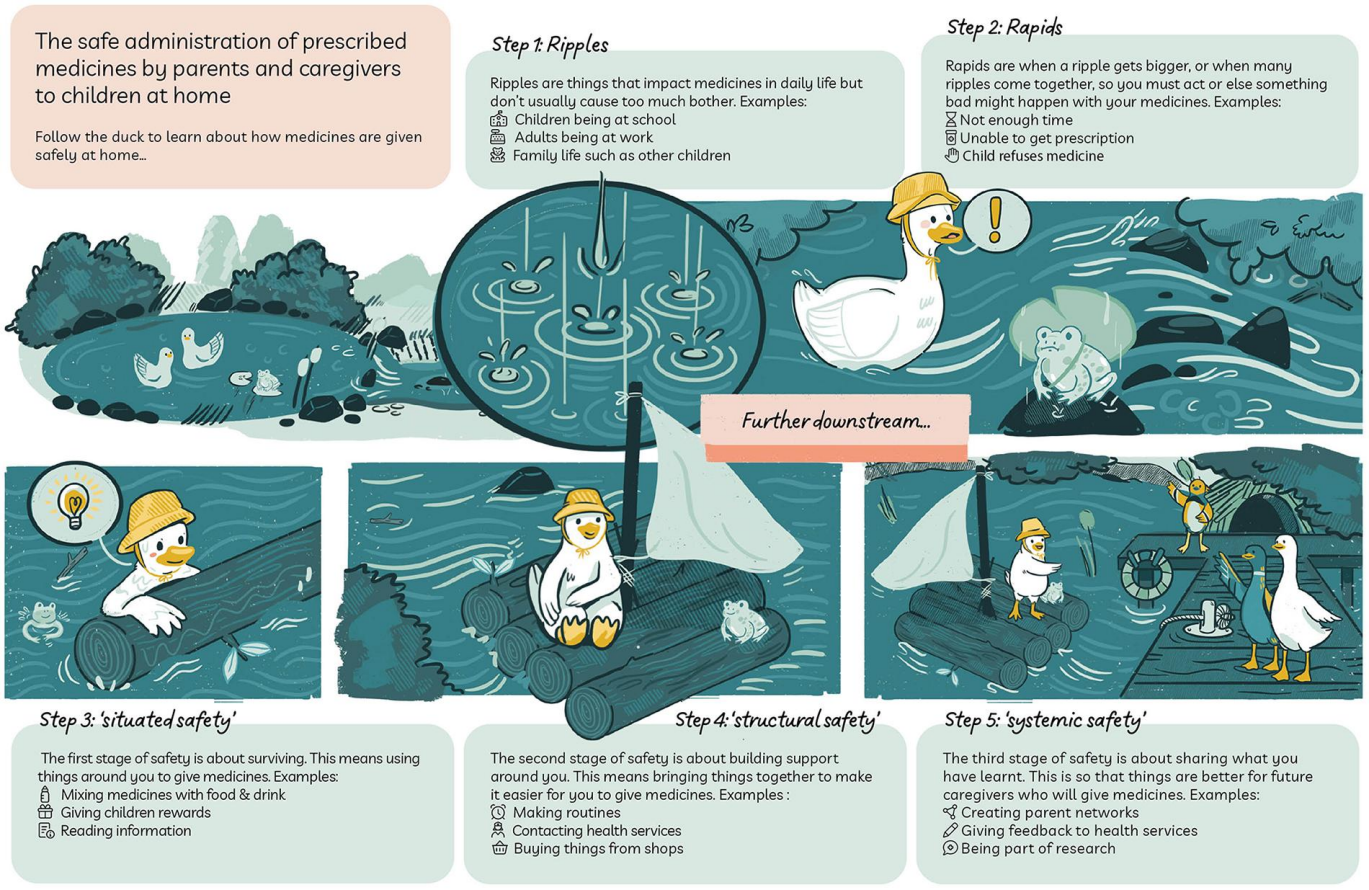
Visual summary of disruptions and adaptations experiences with examples.

A visual summary (see Figure 2: Visual summary of disruptions and adaptations experiences with examples) has also been produced to summarise the key findings.

#### 3.4.1 Descriptions of fluctuations

Parents and caregivers clearly described experiences within studies that could be categorised as fluctuations (variations in everyday life that did not pose an immediate threat to situated control). These fluctuations were a significant part of their lived experience and were described in relation to medicines specifically, or the context in which they were being used (i.e., within family life). Caregivers described this as a contradictory experience at times, depending on whether it was the safe use of medicines or family life which they needed to control (41, 46, 51, 75, 79, 81).

“Juggling family responsibilities and treatment was a constant balancing act” Researcher (81)

Some examples of how family life contributed to fluctuations included: employment (37, 40, 43), partners (36), siblings (36, 40, 83), pets, (83) school (33, 83), extracurricular activities (e.g., sports clubs) (36, 83), bedtimes (66), and domestic work (46, 83).

Children themselves were frequently cited as a source of fluctuation, particularly when resistant to taking medicines (36, 49, 50, 58, 60, 75, 79, 83).

“She never wants to take it.” Participant (79)

Medicines and equipment contributed to fluctuations when poorly designed for their intended purpose (34, 48, 70, 74, 78). For example, the lack of liquid medicines with acceptable flavours for children (37, 55, 58, 69, 71, 79).

“the [medicine] has a very bad taste and he does not like it” Participant (71)

Other fluctuations were related to language and communication, occurring where caregivers and professionals were not fluent in the same language (42, 46, 49), or from differences in understanding of technical language (43, 46, 63, 81, 83).

“the terms antibiotics, bacteria and viruses have been adapted from English and brought into the Kiswahli language” Researcher (46)

#### 3.4.2 Descriptions of disruptions

Disruptions were commonly described around as discreet instances where action was required to avoid loss of control. Disruptions resulted from either a single fluctuation that had escalated, or from multiple interacting fluctuations.

Disruptions were described before the administration of medicines could commence. For example, scheduling the correct time that a medicine is needed and being available to administer.

“When I go to work, I ask for help to give her the dose” Participant (79)

Further ‘pre-administration’ disruptions were also experienced from systems controlling access to necessary resources to administer prescribed medicines (32, 34, 53, 59).

“General Practitioner declining to prescribe…unavailability at community pharmacy … difficulty with repeat prescription process.” Researcher (34)

Once the process of administration began, immediate disruptions were described where medicines or other supplied equipment failed to function successfully (33, 39, 42, 53, 58, 64, 67, 78, 79, 83).

“Her dosage is 6.1 mL on her prescription, but I can’t get 6.1 mL with a syringe.” Participant (42)

As the process of administration continued, further disruptions were described from children who refused to adhere with the administration of the medicine using either verbal or physical signs (43, 48, 59, 60, 61, 69).

“It’s such a nightmare to get her to take them she fights us and scrunches her eyes so tight there is no way the drops go in” Participant (60)

And finally, after a medicine was administered, caregivers described the need to remain vigilant for further disruptions. The most notable example being children's tendency to vomit or spit out doses (41, 43, 50, 51, 69, 79).

“Every time we’d give it to her, she’d throw it up” Participant (69)

#### 3.4.3 Descriptions of situated adaptations

Descriptions of adaptations began with parents and caregivers, and primarily served to focus their attention on the process required to administer medicines safely. An example of this was being conscious of time in order to avoid overdosing or missing doses (34, 41, 61, 63, 74).

“we were watching the clock right to the minute as far as when we gave her the medication” Participant (61)

This situational awareness included adaptations for co-ordinating what tasks had been completed, and what was scheduled to be done (32, 33, 43).

“I take all the bottles out of the cupboard and line them up, but when I’ve drawn the first one, I close the bottle, put it back in the box and put it in the cupboard” Participant (33).

Additional awareness was needed to identify unexpected fluctuations that could escalate to disruptions.

“Parents described how they pay attention to whether their children’s medications were consistent in shape, colour, size and manufacturer.” Researcher (63)

Other caregivers also contributed to adaptations in the situated context, but were required to be immediately available, such as partners or extended family members living in the same location (34, 36, 59, 60, 67, 83).

“South Asian families were more likely to have extended family living with them, and these relatives were more likely to have a role in caring for a child.” Researcher (59)

Other than adaptations to caregivers, additional adaptations involved using available resources in the home. This included food and drink (44, 52, 54, 79), cutlery (39, 50, 64, 78), household items (e.g., towels) (34, 60, 77), household fixtures (e.g., kitchen tops and curtain rails) (33, 67), and pre-supplied equipment (64).

“some used scissors to open capsules or a knife to split tablets” Researcher (39)

These immediately available resources were influenced by local factors such as infrastructure (50) and culture (59, 78). For example, items that are commonly used during daily life within communities.

“Parents noted using an infant feeding device (commonly known as Gokarna/paladai in India). They transferred the medicines from the spoon into the feeding device and then administered.” Researcher (78)

Disruptions related to medicines had clearly described adaptations related to those medicines, either to achieve the correct dose (e.g., splitting tablets, diluting liquids) (33, 55, 72, 74, 78), or actions to improve the acceptability (e.g., improving flavour) (34, 44, 49, 52, 54, 79).

“Caregivers described utilizing various strategies to ensure the full dose of a medication is administered, such as crushing or cutting pills in portions.” Researcher (63)

Despite these adaptive actions described by caregivers to medicines, further encouragement was often needed to facilitate medicines administration. Examples include rewards (36, 49, 60, 79), distractions (34, 52, 63, 64, 81), role play, (34, 81) and covert administration (44, 54, 62, 79).

“many respondents engaged in open (e.g., rewards, play, persuasions, discussion) or hidden (e.g., camouflage, asleep, distraction) techniques.” Researcher (79)

However, another notable adaptation described to children was physical restraint. Often used with young children, or as a last resort to facilitate administration when other adaptations had failed. Techniques for restraint included holding hands or pinning down (49–51, 60, 65, 69).

“As soon as he sees [the medicine] he tries to run away, but then I just grab him.” Participant (69)

Following administration, strategies could be used to ensure the complete dose was given and that any side effects are managed accordingly (33, 43, 63). For example, keeping an infant sitting upright after administration to stop them from spitting the dose out (63).

Information was also accessed in order to learn how to deal with disruptions. This varied in terms of its type [e.g., written (32, 37, 39, 63, 68, 82), verbal, (37, 49, 67, 75), or video (83)], source [e.g., healthcare professional (32, 37, 46, 49, 59, 61, 63, 67, 78, 83), other parent or caregiver, (46, 49, 63, 68) or extended families members (37, 59)] and access route [e.g., in person (49, 59), telephone (51, 59, 61, 67), or online (32, 39, 48, 63, 68)].

“written information was good, because I could refer back to it if it was a week or two since I had done it, to jog my memory.” Participant (67)

Overall, the descriptions of situated adaptations was frequent across studies and were consistent with the definition of immediate mobilisation of easily accessible resources.

#### 3.4.4 Descriptions of structural adaptations

Structural adaptions were described extensively throughout the studies and included reorganising activities and resources to manage any frequent or severe disruptions, or to alleviate the need for ongoing situated adaptations.

The most notable structural adaptation was where parents and caregivers increased the amount of physical or mental capacity they had to care for their child. For example, by sacrificing responsibilities such as employment or social activities (33, 44, 45, 47, 61, 76).

“[Mothers] often give up their personal life to dedicate themselves to caring for their child.” Researcher (76)

This extended to other family members or members of the community wo offered to help (32, 37, 49, 51, 59, 61, 65, 69, 71, 80, 83).

“She [grandmother] helped out a lot.” Participant (51)

Caregivers described how the development and enforcing of routines to organise tasks, resources and people became a central strategy for using medicines safely (36, 37, 39, 41, 44, 51, 55, 62, 63, 72, 74, 79, 81).

“The routine was an adaptation of their everyday life, including administering drugs at specific hours, planning meals and bedtime, preparing doses in advance, and managing the treatment calendar.” Researcher (41)

Further examples of restructured resources included setting alarms to provide reminders for when medicines where due (41, 63, 79), using charts or diaries to record medicines administration (33, 34, 63, 66, 79), modifying the home environment (e.g., putting hooks on walls to hold equipment) (67, 83), and using materials (e.g., baskets) to organise tasks (32, 34, 44).

“These strategies were supported by a wide range of practices (e.g., colour coding and annotation), visual cues (e.g., specific locations in the household) and artefacts such as storage devices, whiteboards, calendars, alarm clocks and smartphone apps.” Researcher (63)

Caregivers described adaptations to how they accessed resources provided by healthcare systems (32–34, 39, 41, 48, 53, 60, 63, 73, 75). For example by finding healthcare professionals could help them find adaptations to disruptions.

“Parents were appreciative of pharmacists who would ‘lend’ small supplies of product when necessary.” Researcher (75)

They also demonstrated structural changes to how they accessed information. This was variable depending on the needs of the caregivers and what was available (34, 41, 59, 60).

“For parents who had difficulties with English, using a local pharmacist who spoke the same language consolidated and clarified information.” Researcher (59)

Caregivers also expanded their social networks to facilitate their access to information and knowledge (59, 63, 68, 78, 81, 83).

“how to get meds into a tiny breastfed baby … I found other parents to talk to about it.” Participant (81)

The majority of structural adaptations related to either caregivers, medicines, or the environment. However, there were descriptions of adaptations to children. For example, by altering any activities the children would otherwise be engaged in (34, 51).

“Quite often I have to keep her off school because they can’t give the new dose.” Participant (34)

Another example was training children with skills to take their medicines to assist caregivers. Specific examples included tablet swallowing (39, 79, 81), and learning about medicines (41, 57).

“most children learned to swallow the tablets.” Researcher (39)

Overall, a great deal of structural adaptations were described, although this was not equal across the people and resources involved.

#### 3.4.5 Descriptions of systemic adaptations

Systemic reform of systems and resources to support the safe use of medicines was experienced by caregivers, but to a lesser extent than situated or structural adaptations. A well described system wide adaptation was the establishment and maintenance of networks to share and distribute resources (e.g., knowledge, medicines, or equipment). This was either described as between caregiver and healthcare provider (48, 51, 59, 61, 75, 81), or between caregivers (55, 56, 63, 66, 81, 83).

“Some [caregivers] actually knew more about resources than their specialists and assumed the role of educating providers.” Researcher (81)

A driving force for caregivers establishing their own networks was where resources provided by healthcare systems were deemed to be insufficient or inaccessible by caregivers. The advent of modern technology, especially digital social media platforms provided a means for caregivers to construct these networks (63, 83).

“I’m also in a support group on Facebook. They’re very sharing and that’s very helpful.” Participant (83)

There was also descriptions of the reform of information to improve communication between professionals and caregivers. For example, using visual methods such as video or images, as opposed to written information (39, 61, 82, 83, 85).

“Many [caregivers] preferred. movie clips where the process of drug handling was visually presented to ensure correct interpretation.” Researcher (39)

There was however little description of reform of medicines themselves or wider systemic changes with healthcare systems.

#### 3.4.6 How do adaptations form during or following disruptions?

Identifying adaptations within studies allowed further exploration of how they developed. Three notable processes were observed at each level of the framework: ‘*trial and error’*, ‘*access to resources’* and ‘*caregiver involvement in design of medicines and systems*’.

##### 3.4.6.1 The formation of situated adaptations - trial and error

The process by which caregivers developed situated adaptations was often referred to as “*trial and error*” (32, 49, 52, 55, 57, 59, 60, 73–75, 77, 79). Caregivers described how an initial disruption triggered the creation and testing of adaptive strategies. Successful adaptations would then be retained after caregivers retrospectively evaluated their effectiveness (33, 54, 57, 63, 68).

“We worked out what worked for us.” Participant (32)

The inclusion of the phrase ‘*error’* also indicates that harm may accompany this process, and this may have negative consequences for caregivers as well as the child.

“Parents described the experience of giving [the medicine] as ‘really quite distressing’ and ‘a horrible, horrible blur’.” Participant quotes with context by Researcher (53)

##### 3.4.6.2 The formation of structural adaptations - access to resources

Access to resources was often cited by caregivers alongside descriptions of structural adaptations. Resources were either readily accessed by caregivers and deployed, or would require access via gatekeepers (i.e., healthcare professionals) before implementation. Examples of readily available resources included household items and items that could be purchased from shops.

“Some participants had purchased oral syringes with caps to carry doses.” Researcher (34)

Lack of access was most notable with prescription only medicines, and this example demonstrates the ’scrambling’ or situated adaptation that results from a lack of access from a gatekeeper (i.e., the prescriber).

“…last minute scrambling to find a prescriber to continue medications.” Researcher (48)

##### 3.4.6.3 The formation of systemic adaptations - caregiver involvement in design of medicines and systems

The clearest description of systemic adaptations was where caregivers were involved in the design of healthcare systems. For example, by learning adaptive techniques and sharing them with healthcare providers, so that changes could be made to resources and systems to avoid disruptions, or so knowledge was passed on to future caregivers about adaptive strategies.

“Iterative changes were made based on parent feedback, including position of IV bags to prevent air in the line.” Researcher (67)

#### 3.4.7 How does research design influence disruptions and adaptations observed?

The mapping of disruptions and adaptations alongside the description of study characteristics revealed an association between the research methods employed and the categories of disruptions and adaptations observed (refer to Table 2: How research design facilitated descriptions of fluctuations, disruptions and adaptations by caregivers).

**Table 2:** How research design facilitated descriptions of fluctuations, disruptions and adaptations by caregivers.

Category	Qualitative Research Design	Example studies
Fluctuations and disruptions	• Open research question and scope (i.e., not focused on a specific healthcare task, e.g., medicines, but on the overall care of a child and family life). • Inclusive research design (e.g., sampling using community venues). • Inductive analysis (e.g., thematic analysis). • Research interview methods that encourage ’situated’ thinking. • Interview guides that asked about ‘problems’.	Doyle, 2022 (33) Lackanpaul, 2017 (59) Benleulmi, 2019 (36) Bracken, 2023 (32)
Situated adaptations	• Small sample with more in-depth interviews. • Conducting interviews at the start or during treatment. • Research interview methods that encourage ’situated’ thinking. • Participant collected data (e.g., internet message board posts).	Law, 2020 (60) Benleulmi, 2019 (36)
Structural adaptations	• Larger sample. • Conducting interviews at the end of treatment. • Interview guides that asked about ‘tips for future parents’. • Focus groups	Camiré-Bernier, 2021 (41) Tang, 2022 (79) Bracken, 2023 (32)
Systemic adaptations	• Studies that either: ○ Followed the entire journey of a patient (e.g., within an treatment pathway), ○ Or, the entire journey of a project (e.g., implementation of home intravenous chemotherapy).	Findlater, 2022 (48) Orford, 2023 (67) Tluczek, 2022 (81)

For example, studies that collected data from caregivers at the end of their child's treatment [e.g., interviews at the end of leukaemia treatment (41, 79)] typically described structural adaptations (e.g., changes to family routines). However, studies that collected data at the start or during treatment [e.g., internet message board posts from caregivers asking for advice regarding eye drop administration (60)] provided descriptions of situated adaptations (e.g., physical restraint).

Interview methodologies also influenced the description of disruptions and adaptations by caregivers. For instance, the use of Vermersch's explicitation interview technique helped caregivers to explore and describe situated actions (36). Other examples include prompting caregivers about ‘*problems’* which helped them to describe fluctuations and disruptions (32), and prompts about ‘*tips for other parents’* which resulted in descriptions of a variety of adaptations (79).

Another important aspect of research was the inclusion of the experiences of a representative sample of caregivers, especially those from communities that are typically underserved by healthcare services. Certain study designs were more effective at including caregivers from these communities, for example by recruiting from a community setting (e.g., community pharmacies) as opposed to large institutions (e.g., hospitals) (59). Disruptions described by these studies was centred around difficulties with accessing healthcare resources, and adaptations included actions to overcome language barriers and use of community specific resources.

## 4 Discussion

This review has provided a comprehensive synthesis of the disruptions and adaptations involved in the safe administration of prescribed medicines to children by parents and caregivers at home. It demonstrates how shortcomings in the design and implementation of medicines and healthcare systems design overall creates disruptions that subsequently impact the safety of children being cared for at home.

Using resilient healthcare theory as a framework has highlighted the significant role that parents and caregivers contribute to safety by identifying potentially harmful disruptions and deploying adaptive strategies where possible to resolve disruptions. This is aligned with safety research undertaken with other patient groups who use medicines or perform other healthcare tasks at home (19–21, 86, 87).

An important finding of this review has been the negative consequences for caregivers from the burden of maintaining safety. Specifically, the fear of causing harm to a child, the use of physical restraint on children, the work involved with creating context specific adaptations, and more generally in the effort involved with the continual deployment of situated adaptations. These factors may provide some explanation as to why caring for a child with a long term health condition has been associated with negative mental health outcomes for caregivers (88). Future inventions to support safe medicines use should include caregiver psychological safety alongside the practical elements of medicines safety.

The relationship between the availability of resources and adaptive capability was clearly described. It is important to acknowledge that access to resources involves social interactions between individuals and the involvement of socially constructed institutions. Therefore, a strength of using a resilient approach is that it is inclusive of these social phenomena, and allows for further exploration of issues of equity, justice and power (89). A notable example from our review was the access of community pharmacy services to overcome language barriers and access knowledge (59). Other studies have also identified that access to resources as an important aspect for caregivers who undertake healthcare roles for a child at home (87, 90). Understanding the relationships between access to resources and health inequities must form a cornerstone for any improvement work in this area.

This review has identified that caregivers try to collaborate with professionals to restructure and reform resources. However, where the involvement of professionals is insufficient or conflicting with their experiences, then caregivers may do this by themselves. This encourages the creation of multiple systems; resulting in duplication, competition and conflict between systems. The advent of digital technologies allows this to occur rapidly and to a greater extent than ever before. Consideration by all professionals groups should be given to how to collaborate effectively with caregivers when designing, implementing and evaluating healthcare systems.

There are currently several medicines safety innovations in development that could reduce disruptions [e.g., 3D-printed tablets (91)] and facilitate the formation of adaptations [e.g calendars for organising medicines administration (92) and smartphone apps (93)]. The important of diverse and high quality qualitative research to inform these innovations should not be overlooked. Several studies included in this review have informed further research (39, 42, 83, 95), and utilising a resilience approach could enhance the potential effectiveness of these interventions or facilitate their implementation.

However, it should be noted that the majority of these reforms are types of systemic adaptations and therefore may take years to develop and implement, and may provide new sources for disruptions. Therefore, the design and provision of resources to support situated and structural adaptations alongside these systemic reforms should not be overlooked.

In conclusion, this review has demonstrated that parents and caregivers undertake a significant amount of work to ensure medicines are used safely at home. Future improvements to healthcare systems should reduce disruptions to avoid the need for adaptations entirely, alongside strengthening the ability of caregivers, professionals and healthcare systems to be adaptive to novel and unpredictable disruptions.

### 4.1 Strengths and limitations

The use of resilient healthcare theory and qualitative synthesis produced an analysis with great breadth and depth. In particular, the inclusion of a range of international and multilingual studies provided a diverse range of cultural values and beliefs. However, some data extracted may have been context specific and not necessarily applicable to all settings. The inclusion of multilingual studies added further depth to this review, but also added the possibility that data were misrepresented or lost during translation.

Studies were ambiguous about their philosophical perspective, failed to acknowledge theoretical and cultural perspectives, and failed to consider how the researchers may have influenced data and findings. The lack of systemic resilience identified may be due to the lack of included studies that have a sufficiently wide scope and duration to identify and describe such processes.

There was a range in participant numbers of included studies. Some studies were comparatively small with regards to participant numbers. However, this may have been a consequence of important methodological considerations with the research design, such as the inclusion of non-English speaking participants, greater emphasis on exploring lived experiences, or the study of sensitive topics such as the use of injectable medicines or enemas. A strength of this review is the inclusion of this diverse set of experiences resulting from different research methodologies.

## Data Availability

The original contributions presented in the study are included in the article/Supplementary Material, further inquiries can be directed to the corresponding author.
