# Supplementary material for: Disruptions to safety and adaptations experienced by parents and caregivers who administered prescribed medicines to children at home: a systematic review using a framework synthesis
Source: Front Health Serv. 2026 Jul 6;6:1748195. doi: 10.3389/frhs.2026.1748195 (PMC13381475; doi:10.3389/frhs.2026.1748195)
Supplement: Supplementary file 1 [file Table1.docx]

**Supplementary Materials**

**Table of Contents**

| **Section** | **Description** | **Page** |
| --- | --- | --- |
| Supplementary Material - Table 1 | ENTREQ Reporting Checklist | 2-5 |
| Supplementary Material - Table 2 | Example Search Strategy | 6 |
| Supplementary Material - Table 3 | Quality Assessment | 7-10 |
| Supplementary Material - Appendix 1 | Public involvement activities | 11-13 |

**Supplementary Material - Table 1: Enhancing transparency in reporting the synthesis of qualitative research (ENTREQ)**

| **No** | **Item** | **Guide and description** | **Line #** |
| --- | --- | --- | --- |
| **1** | Aim | State the research question the synthesis addresses. | Abstract: 12  Main document: 120 |
| **2** | Synthesis methodology | Identify the synthesis methodology or theoretical framework which underpins the synthesis, and describe the rationale for choice of methodology *(e.g. meta-ethnography, thematic synthesis, critical interpretive synthesis, grounded theory synthesis, realist synthesis, meta-aggregation, meta-study, framework synthesis).* | 123 onwards |
| **3** | Approach to searching | Indicate whether the search was pre-planned (*comprehensive search strategies to seek all available studies)* or iterative (*to seek all available concepts until they theoretical saturation is achieved)*. | 152 |
| **4** | Inclusion criteria | Specify the inclusion/exclusion criteria *(e.g. in terms of population, language, year limits, type of publication, study type).* | 160 |
| **5** | Data sources | Describe the information sources used (e.g. *electronic databases (MEDLINE, EMBASE, CINAHL, psycINFO, Econlit), grey literature databases (digital thesis, policy reports), relevant organisational websites, experts, information specialists, generic web searches (Google Scholar) hand searching, reference lists)* and when the searches conducted; provide the rationale for using the data sources. | 153 |
| **6** | Electronic Search strategy | Describe the literature search *(e.g. provide electronic search strategies with population terms, clinical or health topic terms, experiential or social phenomena related terms, filters for qualitative research, and search limits)*. | 155 and Supplementary Materials Table 2 |
| **7** | Study screening methods | Describe the process of study screening and sifting *(e.g. title, abstract and full text review, number of independent reviewers who screened studies).* | 167 |
| **8** | Study characteristics | Present the characteristics of the included studies *(e.g. year of publication, country, population, number of participants, data collection, methodology, analysis, research questions).* | 223 |
| **9** | Study selection results | Identify the number of studies screened and provide reasons for study exclusion *(e,g, for comprehensive searching, provide numbers of studies screened and reasons for exclusion indicated in a figure/flowchart; for iterative searching describe reasons for study exclusion and inclusion based on modifications t the research question and/or contribution to theory development).* | Main document: 200 and Figure 1 PRISMA diagram on 214 |
| **10** | Rationale for appraisal | Describe the rationale and approach used to appraise the included studies or selected findings *(e.g. assessment of conduct (validity and robustness), assessment of reporting (transparency), assessment of content and utility of the findings).* | 175 and Supplementary Materials Table 3. |
| **11** | Appraisal items | State the tools, frameworks and criteria used to appraise the studies or selected findings *(e.g. Existing tools: CASP, QARI, COREQ, Mays and Pope* [[25](https://bmcmedresmethodol.biomedcentral.com/articles/10.1186/1471-2288-12-181#ref-CR25)]*; reviewer developed tools; describe the domains assessed: research team, study design, data analysis and interpretations, reporting).* | 175 and Supplementary Materials Table 3. |
| **12** | Appraisal process | Indicate whether the appraisal was conducted independently by more than one reviewer and if consensus was required. | 175 |
| **13** | Appraisal results | Present results of the quality assessment and indicate which articles, if any, were weighted/excluded based on the assessment and give the rationale. | Supplementary Materials Table 3. |
| **14** | Data extraction | Indicate which sections of the primary studies were analysed and how were the data extracted from the primary studies? *(e.g. all text under the headings “results /conclusions” were extracted electronically and entered into a computer software).* | 184 |
| **15** | Software | State the computer software used, if any. | 168 – Rayyan  192 - ChatGPT  185 - Microsoft Edge PDF editor  188 - Microsoft Excel |
| **16** | Number of reviewers | Identify who was involved in coding and analysis. | 222 and Acknowledgements |
| **17** | Coding | Describe the process for coding of data *(e.g. line by line coding to search for concepts).* | 184 |
| **18** | Study comparison | Describe how were comparisons made within and across studies *(e.g. subsequent studies were coded into pre-existing concepts, and new concepts were created when deemed necessary).* | 188 |
| **19** | Derivation of themes | Explain whether the process of deriving the themes or constructs was inductive or deductive. | 132 |
| **20** | Quotations | Provide quotations from the primary studies to illustrate themes/constructs, and identify whether the quotations were participant quotations of the author’s interpretation. | 232 onwards  Quotations labelled appropriately. |
| **21** | Synthesis output | Present rich, compelling and useful results that go beyond a summary of the primary studies (e.g. *new interpretation, models of evidence, conceptual models, analytical framework, development of a new theory or construct).* | 232 onwards. |

**Supplementary Material – Table 2: Example search strategy**

|  | Concept | Search Strategy | Totals |
| --- | --- | --- | --- |
| #1 | Parent or carer | (exp parent/ OR parent$.ti,ab. OR exp legal guardian/ OR exp caregiver/ OR caregive*.ti,ab. OR mother$.ti,ab. OR father$.ti,ab.) | 1216021 |
| #2 | Medicine administration | (exp prescription/ OR prescription$.ti,ab. OR exp drug therapy/ OR (giv* adj4 medic*).ti,ab. OR (medic* adj4 administ*).ti,ab. OR (drug adj4 administ*).ti,ab.) | 4102746 |
| #3 | At home | (exp primary health care/ OR exp home environment/ OR exp home care services/ OR home*.ti,ab. OR house*.ti,ab.) | 1398748 |
| #4 | - | #1 AND #2 AND #3 | 11040 |

Database: Embase Classic+Embase <1947 to 2024 March 04>

Date of search: March 14^th^ 2024

Total retrieved results: 11040

**Supplementary Material – Table 3: Quality assessment of included studies**

| **Lead Author and Date** | **1. Is there congruity between the stated philosophical perspective and the research methodology?** | **2. Is there congruity between the research methodology and the research question or objectives?** | **3. Is there congruity between the research methodology and the methods used to collect data?** | **4. Is there congruity between the research methodology and the representation and analysis of data?** | **5. Is there congruity between the research methodology and the interpretation of results?** | **6. Is there a statement locating the researcher culturally or theoretically?** | **7. Is the influence of the researcher on the research, and vice- versa, addressed?** | **8. Are participants, and their voices, adequately**  **represented?** | **9. Is the research ethical according to current criteria or, for recent studies, and is there evidence of ethical**  **approval by an appropriate body?** | **10. Do the conclusions drawn in the research report flow from the analysis, or interpretation, of the data?** |
| --- | --- | --- | --- | --- | --- | --- | --- | --- | --- | --- |
| Akkawi, 2023 (1) | N ⚫ | Y ⚫ | Y ⚫ | Y ⚫ | Y ⚫ | N ⚫ | N ⚫ | Y ⚫ | Y ⚫ | Y ⚫ |
| Arnold, 2018 (2) | N ⚫ | Y ⚫ | Y ⚫ | Y ⚫ | Y ⚫ | N ⚫ | N ⚫ | Y ⚫ | Y ⚫ | Y ⚫ |
| Aston, 2018 (3) | N ⚫ | Y ⚫ | Y ⚫ | Y ⚫ | Y ⚫ | N ⚫ | U ⚫ | Y ⚫ | Y ⚫ | Y ⚫ |
| Benlulmi, 2019 (4) | Y ⚫ | Y ⚫ | Y ⚫ | Y ⚫ | Y ⚫ | N ⚫ | N ⚫ | Y ⚫ | U ⚫ | Y ⚫ |
| Bracken, 2023 (5) | U ⚫ | Y ⚫ | U ⚫ | Y ⚫ | Y ⚫ | N ⚫ | N ⚫ | Y ⚫ | Y ⚫ | Y ⚫ |
| Camiré-Bernier, 2021 (6) | N ⚫ | Y ⚫ | Y ⚫ | Y ⚫ | Y ⚫ | N ⚫ | N ⚫ | Y ⚫ | Y ⚫ | Y ⚫ |
| Carroll, 2023 (7) | N ⚫ | U ⚫ | U ⚫ | U ⚫ | U ⚫ | N ⚫ | N ⚫ | N ⚫ | U ⚫ | Y ⚫ |
| Chew, 2020 (8) | N ⚫ | Y ⚫ | Y ⚫ | U ⚫ | U ⚫ | N ⚫ | N ⚫ | Y ⚫ | Y ⚫ | Y ⚫ |
| Coelho, 2015 (9) | U ⚫ | Y ⚫ | Y ⚫ | Y ⚫ | Y ⚫ | N ⚫ | N ⚫ | Y ⚫ | Y ⚫ | Y ⚫ |
| Doyle, 2022 (10) | Y ⚫ | Y ⚫ | Y ⚫ | Y ⚫ | Y ⚫ | N ⚫ | N ⚫ | U ⚫ | Y ⚫ | Y ⚫ |
| Dy, 2018 (11) | N ⚫ | U ⚫ | U ⚫ | U ⚫ | U ⚫ | N ⚫ | U ⚫ | Y ⚫ | Y ⚫ | Y ⚫ |
| Emgård, 2022 (12) | Y ⚫ | Y ⚫ | U ⚫ | U ⚫ | Y ⚫ | U ⚫ | Y ⚫ | Y ⚫ | Y ⚫ | Y ⚫ |
| Fang, 2024 (13) | Y ⚫ | Y ⚫ | Y ⚫ | Y ⚫ | Y ⚫ | N ⚫ | N ⚫ | Y ⚫ | Y ⚫ | Y ⚫ |
| Findlater, 2022 (14) | N ⚫ | Y ⚫ | Y ⚫ | Y ⚫ | Y ⚫ | N ⚫ | N ⚫ | Y ⚫ | Y ⚫ | Y ⚫ |
| Flankegård, 2020 (15) | Y ⚫ | Y ⚫ | Y ⚫ | Y ⚫ | Y ⚫ | N ⚫ | U ⚫ | Y ⚫ | Y ⚫ | Y ⚫ |
| Flynn, 2019 (16) | U ⚫ | Y ⚫ | N ⚫ | U ⚫ | Y ⚫ | U ⚫ | U ⚫ | Y ⚫ | Y ⚫ | Y ⚫ |
| Forsner, 2014 (17) | N ⚫ | Y ⚫ | Y ⚫ | Y ⚫ | Y ⚫ | N ⚫ | N ⚫ | Y ⚫ | Y ⚫ | Y ⚫ |
| Gedaly-Duff, 1994 (18) | Y ⚫ | Y ⚫ | Y ⚫ | Y ⚫ | Y ⚫ | N ⚫ | N ⚫ | Y ⚫ | U ⚫ | Y ⚫ |
| Gilmore, 2022 (19) | N ⚫ | Y ⚫ | Y ⚫ | Y ⚫ | Y ⚫ | N ⚫ | N ⚫ | Y ⚫ | Y ⚫ | Y ⚫ |
| Gomes, 2019 (20) | Y ⚫ | Y ⚫ | Y ⚫ | Y ⚫ | Y ⚫ | Y ⚫ | U ⚫ | Y ⚫ | Y ⚫ | Y ⚫ |
| Hoegy, 2019 (21) | U ⚫ | Y ⚫ | Y ⚫ | Y ⚫ | U ⚫ | N ⚫ | U ⚫ | Y ⚫ | Y ⚫ | Y ⚫ |
| King, 2018 (22) | U ⚫ | Y ⚫ | Y ⚫ | Y ⚫ | Y ⚫ | Y ⚫ | Y ⚫ | Y ⚫ | Y ⚫ | Y ⚫ |
| Klok, 2014 (23) | N ⚫ | Y ⚫ | U ⚫ | U ⚫ | U ⚫ | N ⚫ | N ⚫ | U ⚫ | Y ⚫ | Y ⚫ |
| Kremeike, 2015 (24) | N ⚫ | Y ⚫ | Y ⚫ | Y ⚫ | Y ⚫ | U ⚫ | N ⚫ | U ⚫ | N ⚫ | Y ⚫ |
| Lakhanpaul, 2017 (25) | U ⚫ | Y ⚫ | U ⚫ | Y ⚫ | Y ⚫ | U ⚫ | N ⚫ | Y ⚫ | Y ⚫ | Y ⚫ |
| Law, 2020 (26) | N ⚫ | Y ⚫ | Y ⚫ | Y ⚫ | Y ⚫ | N ⚫ | N ⚫ | Y ⚫ | Y ⚫ | Y ⚫ |
| Longard, 2016 (27) | U ⚫ | Y ⚫ | Y ⚫ | Y ⚫ | Y ⚫ | N ⚫ | U ⚫ | Y ⚫ | Y ⚫ | Y ⚫ |
| McCall, 2017 (28) | N ⚫ | Y ⚫ | Y ⚫ | Y ⚫ | Y ⚫ | N ⚫ | N ⚫ | Y ⚫ | Y ⚫ | Y ⚫ |
| Min, 2023 (29) | U ⚫ | Y ⚫ | Y ⚫ | Y ⚫ | Y ⚫ | N ⚫ | N ⚫ | Y ⚫ | U ⚫ | Y ⚫ |
| Monnerat, 2016 (30) | Y ⚫ | Y ⚫ | Y ⚫ | Y ⚫ | Y ⚫ | N ⚫ | N ⚫ | Y ⚫ | Y ⚫ | Y ⚫ |
| Ndou, 2022 (31) | N ⚫ | Y ⚫ | Y ⚫ | Y ⚫ | Y ⚫ | Y ⚫ | Y ⚫ | Y ⚫ | Y ⚫ | Y ⚫ |
| Okido, 2016 (32) | U ⚫ | Y ⚫ | Y ⚫ | Y ⚫ | Y ⚫ | N ⚫ | N ⚫ | Y ⚫ | Y ⚫ | Y ⚫ |
| Orford, 2023 (33) | N ⚫ | Y ⚫ | Y ⚫ | U ⚫ | Y ⚫ | N ⚫ | N ⚫ | Y ⚫ | N ⚫ | Y ⚫ |
| Phillips, 2023 (34) | U ⚫ | Y ⚫ | Y ⚫ | Y ⚫ | Y ⚫ | N ⚫ | U ⚫ | Y ⚫ | Y ⚫ | Y ⚫ |
| Powers, 2020 (35) | U ⚫ | Y ⚫ | Y ⚫ | U ⚫ | U ⚫ | N ⚫ | N ⚫ | Y ⚫ | Y ⚫ | Y ⚫ |
| Rahimi, 2019 (36) | U ⚫ | Y ⚫ | Y ⚫ | Y ⚫ | Y ⚫ | N ⚫ | U ⚫ | Y ⚫ | Y ⚫ | Y ⚫ |
| Ramos, 2018 (37) | U ⚫ | Y ⚫ | Y ⚫ | Y ⚫ | Y ⚫ | N ⚫ | N ⚫ | Y ⚫ | Y ⚫ | Y ⚫ |
| Ribeiro, 2022 (38) | U ⚫ | Y ⚫ | Y ⚫ | U ⚫ | Y ⚫ | N ⚫ | N ⚫ | Y ⚫ | Y ⚫ | Y ⚫ |
| Santer, 2012 (39) | N ⚫ | Y ⚫ | Y ⚫ | Y ⚫ | Y ⚫ | N ⚫ | N ⚫ | Y ⚫ | Y ⚫ | Y ⚫ |
| Schmitd, 2012(40) | Y ⚫ | Y ⚫ | U ⚫ | Y ⚫ | Y ⚫ | N ⚫ | N ⚫ | Y ⚫ | Y ⚫ | Y ⚫ |
| Silveira, 2022 (41) | N ⚫ | Y ⚫ | Y ⚫ | Y ⚫ | Y ⚫ | N ⚫ | N ⚫ | Y ⚫ | Y ⚫ | Y ⚫ |
| Silver-Rodrigues, 2018 (42) | N ⚫ | Y ⚫ | Y ⚫ | Y ⚫ | Y ⚫ | N ⚫ | N ⚫ | U ⚫ | Y ⚫ | Y ⚫ |
| Slatter, 2004 (43) | U ⚫ | Y ⚫ | Y ⚫ | U ⚫ | Y ⚫ | U ⚫ | N ⚫ | U ⚫ | Y ⚫ | Y ⚫ |
| Souza, 2020 (44) | N ⚫ | Y ⚫ | Y ⚫ | Y ⚫ | Y ⚫ | N ⚫ | N ⚫ | Y ⚫ | Y ⚫ | Y ⚫ |
| Sutton, 2022 (45) | N ⚫ | U ⚫ | Y ⚫ | Y ⚫ | Y ⚫ | N ⚫ | N ⚫ | U ⚫ | Y ⚫ | Y ⚫ |
| Talegaonkar, 2023 (46) | U ⚫ | Y ⚫ | Y ⚫ | Y ⚫ | Y ⚫ | N ⚫ | N ⚫ | Y ⚫ | Y ⚫ | Y ⚫ |
| Tang, 2022 (47) | N ⚫ | Y ⚫ | Y ⚫ | U ⚫ | U ⚫ | N ⚫ | N ⚫ | U ⚫ | Y ⚫ | Y ⚫ |
| Tran, 2017 (48) | N ⚫ | Y ⚫ | Y ⚫ | Y ⚫ | Y ⚫ | N ⚫ | N ⚫ | Y ⚫ | Y ⚫ | Y ⚫ |
| Tulczek, 2022 (49) | N ⚫ | U ⚫ | U ⚫ | U ⚫ | U ⚫ | U ⚫ | U ⚫ | U ⚫ | Y ⚫ | Y ⚫ |
| van Uum, 2019 (50) | N ⚫ | U ⚫ | U ⚫ | U ⚫ | U ⚫ | U ⚫ | Y ⚫ | Y ⚫ | Y ⚫ | Y ⚫ |
| Zupanec 2025 (51) | N ⚫ | Y ⚫ | Y ⚫ | U ⚫ | Y ⚫ | N ⚫ | N ⚫ | U ⚫ | Y ⚫ | Y ⚫ |

Key: Y = Yes, N ⚫ = No, U ⚫ = Unsure

**Supplementary Material – Appendix 1: Public involvement activities**

**Overview of Activities**

This review took an iterative approach to public involvement depending on the stage of the research. Public involvement has been conducted using 4 types of activities:

- conducting a focus group,
- meeting individual public advisors,
- establishing and running meetings for a project specific public advisory group,
- and attendance at community led events.

A summary of how the public have been involved and their impact on the review can be seen in Table 1.

**Table 1: Summary of public involvement activities in relation to stage of systematic review**

| **Stage** | **Public Involvement Activity** | **Influence on Research** |
| --- | --- | --- |
| Research design - Research Question | Focus Group  Community engagement | Setting question.  Helping identify important outcomes.  Understanding lived experience. |
| Research design - Theoretical Approach | Parent advisory group #1  Parent advisors | Helping explore the feasibility of theoretical approach. |
| Research design - Funding | Parent advisors | Sharing digital photos of lived experience.  Reviewing funding applications.  Reviewing Plain English Summary. |
| Data analysis – data extraction | Parent advisors | Helping to complete co-extraction of papers with researcher. |
| Data analysis - interpretation | Parent advisory group #2 | Testing and refining framework. |
| Data analysis - triangulation | Community engagement | Checking relevance of data extracted from international studies to UK families. |
| Results | Parent advisors | Reviewing early drafts and providing feedback. |

These activities have influenced the research in the following ways:

- Study design – research question. Advisors helped with identifying and setting a relevant during a focus group. This was refined with further engagement at community events.
- Study design – theoretical approach. Individual advisors have given feedback about the feasibility of using a patient safety approach.
- Study design – funding. Individual advisors provided stories and imagery that were used as part of the successful funding application.
- Data analysis – data extraction. Individual advisors helped extract data from studies that was used in the review.
- Data analysis – interpretation. Individual advisors and the public advisory group reviewed extracted data and helped
- Data analysis – triangulation. Community engagement events helped to provide perspectives for the research and relevance to their lived experiences.
- Results – producing manuscripts. Individual advisors have reviewed and edited manuscripts to ensure readability and accessibility of the findings of the review.

**Details of Focus Group – 22^nd^ October 2019**

Recruitment:

There was no parent involvement groups at our institution, and therefore we advertised in multiple places to ensure we could recruit enough participants. An A5 flyer was written to summarise the key information. Participants were recruited from several sources: social media, neonatal unit annual celebration event, outpatient clinics, veteran parents circulated through their networks and specialist Nurses forwarded advert to parents.

Participants:

5 mothers.

Activities:

The group were asked to discuss the following questions:

1. Talk about your experience of having to give medicines at home. What problems did you have? Was everything fine? What went well? How did you feel about taking responsibility for giving medicines?
2. Talk about the effect of any problems (or things that went well)? How did it make you feel? What was the effect on your child? What did you do to resolve the problem? Did you visit the GP, or hospital to sort it out? Did you search for information (e.g. internet, friend, social media)?
3. Talk about what could have helped? What would you do different a second time? What did you learn that was helpful? Who did you find that was helpful?

Findings:

The main outcome was that the feedback focussed the project on medication safety. This has helped to narrow the research question and ensure it is relevant to current priorities for families, and the wider NHS.

**Details of Parent Advisory Group #1 – 29^th^ June 2024**

Recruitment:

Families were recruited through existing contacts, in person events (e.g. world prematurity day events at our local institution) and online forums (neonatal facebook groups, twitter, etc.).

Participants:

In total, 5 families attended the event. They comprised of 1 father/mother dyad, 5 mothers and 5 children (totalling 11 participants in total). To help run the event a play worker organised activities for the children.

Activities:

The first half of the meeting was a guided discussion using the following prompts:

- What does the term resilience mean to me?
- How have I experienced resilience?

Participants were asked to write down their interpretation of resilience on a post-IT note, and then discuss in pairs their experiences of resilience. Each pair was then asked to feedback their discussion to the group. A brief discussion was then had between the whole group.

Findings:

The group agreed that resilience was a good concept to ‘standardise’ experience and found it useful to relate to each other’s experiences. The meeting demonstrated that using the concept of resilience was acknowledged by participants as acceptable and they were able to relate it to their lived experiences.

**Details of Parent Advisory Group #2 – 25^th^ January 2025**

Recruitment:

Participants were invited from the 1^st^ meeting with additional adverts being circulated as previously described.

Participants:

In total, 5 families attended the event. They comprised of 1 father/mother dyad, 1 father, 4 mothers and 6 children (totalling 12 participants in total). There was also a graduate student and play worker who helped facilitate the event if you participated along with the lead researcher. The play worker organised activities for the children who were too young to participate.

Activities:

The group completed an activity that was divided into two parts:

1. The first half of the meeting was a structured activity on the topic of storytelling involving adversity at home. Participants were asked to reflect on a problem that they have had to solve at home. After a short time of reflection and writing, participants were encouraged to group into pairs to discuss each other’s stories with one another. Participants were encouraged to add details according to questions asked by their group. After another period of 15 minutes, each of the four pairs shared one of their stories with the group.
2. After a short break in the second half of the meeting the group took their stories and tried to isolate the individual disruptions within them. Participants were then encouraged to write down these individual disruptions on post-it notes and add them to a timeline on the wall.

Findings:

Reviewing the post it notes showed that the parents were able to isolate particular things from their stories. The way they were added to the timeline did show some resemblance of a chronological order. For example, towards the beginning there are disruptions due to accessing services. And towards the end there are more practical elements of care such as needing to travel to access resources administer medicines.

From the comments it’s also clear that parents were able to primarily pick out disruptions. The longer comments did start to identify the adaptive behaviours that then followed. On the whole the activity appears to work quite well at keeping the advisors on topic. There were some discordant parts of this activity. For instance, vomiting was mentioned at several points. There was also a missed opportunity for a better discussion of what the participants had written.

Overall the activity was useful to help with mapping and interpretation of the systematic review findings.
